# THE RELATIONSHIP BETWEEN CREATININE-TO-CYSTATIN C RATIO AND FRAILTY: THE MEDIATING ROLE OF MUSCLE STRENGTH

**DOI:** 10.1093/geroni/igae098.4091

**Published:** 2024-12-31

**Authors:** Shuli Jia, Mei-Ling Ge, Qian-Li Xue, Bigrong Dong

**Affiliations:** West China Hospital, Sichuan University, Chengdu, Sichuan, China (People’s Republic); West China Hospital, Sichuan University, Chengdu, Sichuan, China (People’s Republic); Johns Hopkins University, Baltimore, Maryland, United States; West China Hospital, Sichuan University, Chengdu, Sichuan, China (People’s Republic)

## Abstract

**Background:**

The creatinine to cystatin C ratio (CCR) has recently been suggested as a predictor for many adverse clinical outcomes. This study aimed to investigate the association between CCR and frailty, as well as the mediating role of muscle strength.

**Methods:**

Cross-sectional and longitudinal analyses with 4-year follow-up of community-dwelling adults aged ≥60 years were conducted. Frailty was assessed using the physical frailty phenotype, which includes five binary criteria (weakness, slowness, exhaustion, low activity level and weight loss). Participants with three or more criteria were defined as frail. Muscle strength was measured by the maximum handgrip strength (HGS) of two hands. Logistic regression was used to analyze the associations between baseline CCR and prevalent and incident frailty. Mediation analyses were performed to examine the mediating role of HGS in the relationship between baseline CCR and incident frailty.

**Results:**

Higher CCR was associated with lower risk of frailty (OR 0.41(0.18,0.92), p=0.030) in cross-sectional analysis, but this relationship was not significant when adding HGS in the regression model. After excluding participants who were frail at baseline and during the mid-follow-up, and those met the criteria for weakness at baseline, a total of 2428 participants were included in the longitudinal analysis. The association between baseline CCR and incident frailty was partially mediated by the mid-follow-up HGS (mediation proportion: 14%, p < 0.001). Conclusions: HGS partially mediated the associations between CCR and frailty in Chinese populations. The findings reveal a novel biomarker for frailty, which may also facilitate the identification of frailty subtypes.

